# Induced and Evoked Human Electrophysiological Correlates of Visual Working Memory Set-Size Effects at Encoding

**DOI:** 10.1371/journal.pone.0167022

**Published:** 2016-11-30

**Authors:** Gennadiy Gurariy, Kyle W. Killebrew, Marian E. Berryhill, Gideon P. Caplovitz

**Affiliations:** University of Nevada, Reno Department of Psychology, Reno, United States of America; University Medical Center Goettingen, GERMANY

## Abstract

The ability to encode, store, and retrieve visually presented objects is referred to as visual working memory (VWM). Although crucial for many cognitive processes, previous research reveals that VWM strictly capacity limited. This capacity limitation is behaviorally observable in the set size effect: the ability to successfully report items in VWM asymptotes at a small number of items. Research into the neural correlates of set size effects and VWM capacity limits in general largely focus on the maintenance period of VWM. However, we previously reported that neural resources allocated to individual items during VWM encoding correspond to successful VWM performance. Here we expand on those findings by investigating neural correlates of set size during VWM encoding. We hypothesized that neural signatures of encoding-related VWM capacity limitations should be differentiable as a function of set size. We tested our hypothesis using High Density Electroencephalography (HD-EEG) to analyze frequency components evoked by flickering target items in VWM displays of set size 2 or 4. We found that set size modulated the amplitude of the 1^st^ and 2^nd^ harmonic frequencies evoked during successful VWM encoding across frontal and occipital-parietal electrodes. Frontal sites exhibited the most robust effects for the 2^nd^ harmonic (set size 2 > set size 4). Additionally, we found a set-size effect on the induced power of delta-band (1–4 Hz) activity (set size 2 > set size 4). These results are consistent with a capacity limited VWM resource at encoding that is distributed across to-be-remembered items in a VWM display. This resource may work in conjunction with a task-specific selection process that determines which items are to be encoded and which are to be ignored. These neural set size effects support the view that VWM capacity limitations begin with encoding related processes.

## Introduction

Visual working memory (VWM) refers to the ability to store and manipulate visual information for brief periods of time. Although VWM is an integral aspect of daily cognition [1,2], it is nonetheless capacity limited [3]. Behaviorally, these limitations clearly reveal themselves when set sizes increase. VWM performance decreases as the encoding, maintenance and retrieval demands increase. A recent focus of cognitive neuroscience research is to understand the neural bases of this capacity limitation. Here, we applied high-density electroencephalography (HD-EEG) to investigate both induced and evoked neural set size effects operating during the encoding phase of VWM.

Prior to objects being stored in VWM, stimuli enter iconic memory. This buffer helps explain the richness of visual experience for brief periods of time as well as transsaccadic perception [4,5]. Although brief (<500ms), iconic memory has a nearly limitless capacity that decays quickly [6–8]. In contrast, VWM is limited to ~4 items [9–11], or fewer [12]. Here, we ask: do limitations arise during VWM encoding?

Set size effects are a hallmark of VWM’s capacity limitation. VWM performance accuracy decreases with an increase in the number of items (set size) presented [13,11]. Recent findings aimed at elucidating the neural bases of VWM capacity limitations have sought to link neural correlates of the behavioral set size effect with stages of VWM [14–17]. Functional magnetic resonance imaging (fMRI) data show that during VWM maintenance the BOLD signal in the intraparietal sulcus (IPS) increases according to the number of items a person is maintaining and this signal reaches asymptote at an individual’s maximum VWM capacity [17–19]. Similarly, EEG research reveals an evoked potential localized to occipito-parietal regions during maintenance of items presented to the contralateral side of the visual field. This physiological signal, known as the contralateral delay activity (CDA), increases in amplitude according to set size and also asymptotes at a participant’s VWM capacity limit [20–22]. To bridge these two literatures, we recently measured fMRI while applying a CDA paradigm. We found that the IPS represents contralaterally presented items in VWM, but that VWM capacity limits did not differ when attending to one or both visual hemifields [23]. These findings provide converging neural evidence that VWM maintenance related processes contribute to VWM capacity limitations.

However, it is clear that the retrieval stage also limits VWM capacity. There is a literature devoted to studying how different task demands elicit different behavioral and neural patterns during retrieval. A prevailing view is that we typically scan through the contents of VWM using serial exhaustive search, even after finding the target item [24–26]. Consequently, with more items in VWM, responses slow, subjecting items in VWM to greater decay and worse performance [27,28].

The conversation thus far overlooks the role encoding plays in limiting VWM capacity: encoding necessarily precedes maintenance and retrieval. Since Miller made ‘chunking’ famous [29], VWM encoding strategy has been considered important [30,31], as is prior knowledge [32]. Behaviorally, encoding duration modulates VWM performance with shorter times leading to less accurate responses during recall [33]. Monkey physiology shows that capacity limitations may emerge during encoding as objects compete for resources that are distributed in a flexible manner within each hemifield [34]. In humans, physiological recordings have shown that the neural activity associated with objects that are physically present is nearly indistinguishable from objects that have been removed from view and are being actively maintained in VWM [35]. These findings confirm the importance of encoding as a contributing factor to capacity limitations.

Recent neural evidence highlighting the importance of encoding comes from studies showing that consistent activations during encoding and maintenance are required for successful retrieval. For example, visual regions (e.g. primary visual cortex) contribute to VWM maintenance in the *absence* of visual stimulation [36,37]. Furthermore, successful VWM performance is predicted by enhanced functional connectivity between extrastriate cortex and lateral prefrontal cortex [38], and between frontoparietal regions [39,40]. Furthermore, functional connectivity shifts predictably depending on set size [41]. Set size also predicts activity in regions associated with long-term memory including hippocampal involvement during encoding [42], and parahippocampal activity during maintenance [43]. It is reasonable to believe that VWM capacity limits arise from each stage of VWM. Yet, questions remain regarding how these limitations emerge.

Recently, we used the classic frequency-tagging EEG technique to examine neural responses to the visual stimuli presented in a VWM task. Frequency-tagging entails rapid and periodic stimulus presentation, inducing corresponding rapid-periodic responses in the simultaneously recorded EEG [44]. We reported that the frequency-tag amplitudes during encoding were significantly larger for successfully remembered items compared to subsequently forgotten items [45]. The frequency-tags of *unprobed* items were also greater on correct trials.

To summarize this technique more specifically, frequency-tags correspond to transient evoked potentials measured at the scalp using EEG. Transforming the EEG signal into the frequency domain allows for the identification of frequency-tags corresponding to *each* flickering stimulus. For example, a stimulus flickering at 5 Hz elicits five potentially superimposed visually evoked potentials per second and thus, produces a 5 Hz signal in the frequency domain. This 5 Hz frequency-tag represents the neural correlate of the stimulus response. In addition to frequency-tags corresponding to the rate of stimulus flicker (fundamental frequency), the EEG also contains harmonics of the fundamental frequency. For example, a stimulus flickering at 5 Hz may elicit 2^nd^ and 3^rd^ harmonics at 10 Hz and 15 Hz, respectively. The fundamental frequency and its harmonics are thought to be driven by different neural populations, the former indicative of bottom-up processing and the latter of top-down processing [46]. Although it is conceivable that a flickering stimulus of sort used in our study may lead to the entrainment of neural ensembles underlying cognitive operations such as VWM encoding, we note that this is not goal of the frequency-tagging procedure. Thus, the stimulus frequencies chosen for the experimental procedure (described in Materials and methods section: Experimental procedure) are arbitrary with regards to endogenous neural oscillations involved in cognitive operations. Rather, we use these frequency-tags to identify neural correlates of *individual stimuli* during VWM encoding.

However, induced oscillations also provide insights regarding cognitive processes, including VWM [47,48]. Unlike evoked oscillations, induced oscillations are not phase-locked to the stimulus and they are associated with cognitive states as well as functional changes in neural processing. These oscillations may reflect changes occurring within as well as between brain structures. Specifically, these changes have been proposed as a mechanism for neural synchronization resulting in short- and long- range communication. Such communication can be cortico-cortical or subcortico-cortical and has been linked to numerous cognitive processes including attention, memory and feature binding [49]. These oscillations can be categorized into the following frequency bands: delta (1–4 Hz), theta (5–8 Hz), alpha (9–12 Hz), beta (12–31 Hz), and gamma (32–100 Hz). For our purposes, frequency-tags are used to investigate the allocation of neural resources to individual items in the VWM paradigm as a function of set size, whereas induced oscillations can be interpreted as a proxy for task-related cognitive processes occurring during the encoding period.

Here, we determined whether neural correlates of set size effects can be observed during VWM encoding exploring both induced and evoked measures. We compared encoding-related neural responses to VWM arrays of set size 2 or 4. We found that during VWM encoding the frequency-tag amplitudes corresponding to successfully retrieved items is modulated by set size. The qualitative nature of this encoding-related neural correlate is distinct from those observed in previous studies examining neural set size effects during maintenance. Unlike fMRI and CDA results showing increases in neural responses with increased set sizes, we find significantly larger neural responses to successfully retrieved items in the 2-item compared to the 4-item set size. In addition, we found an effect of set-size on the amplitude of the induced delta-band activity. Again, increased power was observed in the set size 2 compared to set size 4. These results are consistent with the conclusion that encoding-related VWM processes are capacity limited and attempts to encode multiple items overtaxes this resource and reduces encoding success.

## Materials and Methods

### Participants

Twenty-four, right handed, neurotypical adults with normal or corrected to normal visual acuity participated (13 male, age 20–40) and provided informed written consent. The Institutional Review Board at the University of Nevada, Reno approved all protocols. As described below, three of these participants were excluded due to excessive artifacts detected in the EEG.

### Stimulus display

Stimuli were displayed on a Mitsubishi Diamond Pro270 CRT monitor (20in, 1024x768) with a 120-Hz refresh rate, running via a 2.6Mhz MacMini and presented using the PsychToolbox [50,51] for MATLAB (MathWorks Inc., Natick, MA). Viewing distance was 57 cm.

### Electrophysiological recordings

The electroencephalogram (EEG) was continuously recorded using a 256 channel HydroCel Geodesic Sensor Net via an EGI Net Amps Bio 300 amplifier (Electrical Geodesics Inc., Eugene, OR) sampling at 1000 Hz. The digital data were recorded using Netstation 5.0(1) software. Impendence values were kept at or below 50 Ω. Frame-accurate timing of stimulus presentation was validated by photodiode.

### Experimental Procedure

**Fig 1:**
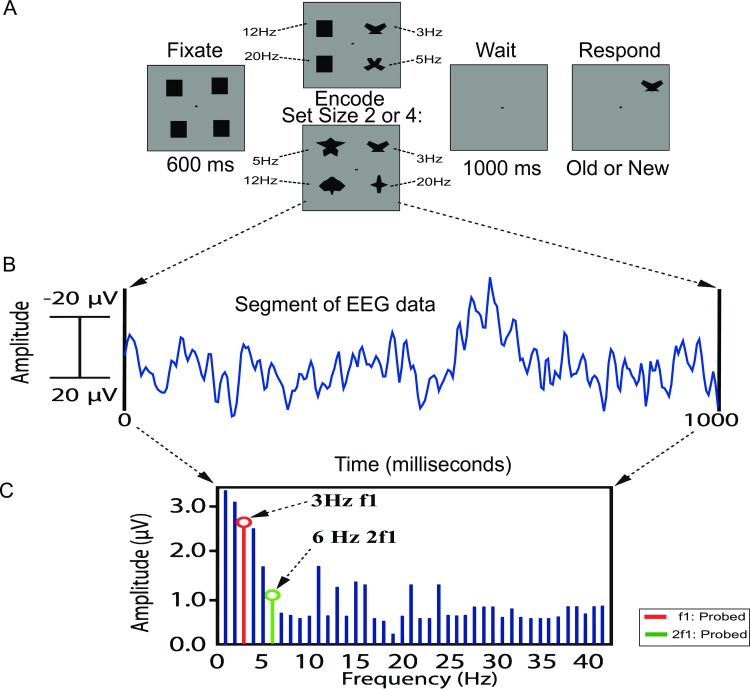
Visual working memory task and data processing sequence. (A) Participants viewed an initial fixation screen (600ms) followed by a memory array (1000ms) in which each item flicked at a distinct frequency (3 Hz, 5 Hz, 12 Hz and 20 Hz). Set size was manipulated to create 2 conditions, set size 2 or set size 4. A delay period (1000ms) followed the stimulus presentation. Finally, a single probed stimulus appeared in one of the four previously presented locations. Participants were instructed to indicate whether the probed stimulus was old (same shape in same location) or new (different shape). *Note*: *black squares were used in the fixation period to control for onset VEP responses that could contaminate the data*. (B) Epochs lasting 1000 ms time-locked to the onset of the memory array were extracted from the raw data. (C) In the evoked analysis, once epochs in each condition were averaged together, a Fourier Transform was applied to the data so that the frequency tag amplitudes could be extracted for the fundamental (red) and harmonic (green) frequencies corresponding to probed items in the stimulus array. Conversely, in the induced condition, the Fourier Transform is applied to each trial prior to averaging.

Participants performed a VWM change detection task with a set size of either 2 or 4 items; see Fig 1. Trials began with fixation (600 ms) consisting of a black central fixation point (0.35° x 0.35°) on a neutral gray background, surrounded by 4 black squares (7° x 7°) that occupied predetermined stimulus positions centered in each of the four quadrants with a random offset of up to 1.5° in any direction. Next, either 2 or 4 square placeholders were replaced by shape stimuli (1000 ms). The shapes were bilaterally symmetrical shapes (7° x 7°) chosen randomly (without replacement) from a set of ten. Stimuli were generated by a previously described algorithm [52,53]. During encoding, each shape reversed contrast (black-white) at a different frequency (3hz, 5hz, 12hz, or 20hz). In the set size 4 condition, each shape flickered at one of the frequencies; in the set size 2 condition, the two shapes flickered at 3hz and 5hz respectively and the two placeholder squares flickered at 12 Hz and 20 Hz. The placeholder squares ensured that any differences between set size 2 and set size 4 were not caused by the flicker of additional stimuli or frequencies in the set size 4 condition. Only the 3Hz and 5 Hz probed trials in the set size 4 condition were analyzed; none of the 12Hz and 20Hz probed items in the set size 4 condition were included in analyses. These specific frequencies were chosen because they allow for an integer number of cycles at the 120hz frame refresh rate during the 1000 ms encoding period, and preserved independence through the third harmonics. The encoding phase was followed by a maintenance period (1000 ms) during which time only the fixation spot was present. Next, a probe stimulus shape reappeared in one quadrant (3000 ms). Participants were instructed to indicate whether this probe item was old (same shape in the same location) or new (different shape or different location) via key press. Thus, correct responses were contingent on accurately maintaining both shape and location. Each frequency (set size 2: 3 Hz, 5 Hz; set size 4: 3 Hz, 5 Hz, 12 Hz & 20 Hz) was probed a total of 76 times (38 were ‘old’, 38 were ‘new’). The entire experiment consisted of 456 trials (set size 2: 152 trials (1/3), set size 4: 304 (2/3)) and 50% of probe items matched what was shown at encoding. Trials were presented in pseudorandom order.

### Electrophysiological data preprocessing

The EEG data were processed using Netstation 5.0 and custom scripts written in MATLAB. A high-pass filter of 0.1 Hz removed slow drift noise. Next, the filtered data was segmented into 1000 ms epochs time-locked to stimulus onset and lasting the duration of the encoding period. Segmentation was done using trigger markers sent from the stimulus computer to the acquisition computer at the onset of every encoding period. The time offset between stimulus appearance and trigger registration was measured using a photodiode and compensated for during trial segmentation. Using Netstation, artifact detection was performed to identify trials containing eye blinks, eye movements, and bad channels; this was followed when possible by bad channel replacement. Trials were marked for exclusion if they contained an eye blink, an eye movement, or >10% bad channels (exclusion criteria on the basis of too many bad trials is discussed in Materials and methods section: Artifact detection and subject exclusion). Using custom MATLAB scripts, the correct trials were grouped into the following 4 conditions based on set size and the flicker frequency of the to-be-probed item: correct 3 Hz/set size 2, correct 5 Hz/set size 2, correct 3 Hz/set size 4, correct 5 Hz/set size 4. The trials probing the 12 Hz or 20 Hz item in the set size 4 condition were excluded from analyses, as were incorrect trials.

### Artifact detection and subject exclusion

As described in the previous section, a trial was marked bad if it contained an eye blink, an eye movement, or if 10% of the channels were found to be bad during the encoding period. Participants whose number of bad trials exceeded 30% were excluded from the study. Under these criteria, 3 people were excluded from the final analysis leaving a total of 21 participants. Among these remaining participants, the mean number of trials per condition was 50.6 with a standard deviation of 8.46. This number takes into account the trials rejected due to artifacts as well as the good trails that were left out during the permutation analysis (see Materials and methods section: Permutation analysis).

### Permutation analysis

Because only correct trials were analyzed there were unequal numbers of trials per condition per participant. To ensure comparable signal-to-noise rations and fairer comparisons between conditions, we conducted a permutation analysis in which an equal number of trials contributed to each condition average. For each participant, we identified the condition with the fewest number of trials and on each iteration of the permutation analysis, randomly selected that number of trials from the other conditions. This process was repeated over multiple iterations (see Materials and methods sections on frequency-tagging and induced power analysis). The trials from each iteration were averaged together appropriately for each of the four conditions: set size: 2 or 4; frequency of probed item: 3 Hz or 5 Hz. Permutation analyses were performed independently for the frequency-tagging and the induced power analysis; these are described below in more detail.

### Frequency-tagging (evoked) analysis

To avoid the random selection of an outlier subset of trials, a total of 50,000 iterations of the permutation analysis were performed and the average across iteration was used as the response to the other conditions. This process resulted in four average time-domain waveforms computed at each electrode for each participant. Each waveform was 1000ms long and corresponded to either the average response for set-size 2 or 4 at the frequency of probed item: 3 Hz or 5 Hz. A Fourier transform was applied to the average of each condition, at each of the 256 channels, so that the frequency-tag amplitudes for the fundamental and second harmonic frequencies could be extracted for the probed items at each set size (2 and 4). Again, the analysis was restricted to the 3Hz and 5Hz trials. For example, if a to-be-probed item flickered at 5 Hz during encoding, then the amplitudes of 5 Hz (1f) and 10 Hz (2f) were used as indices for neural activity related to VWM encoding of the to-be-probed item.

At each electrode site, a set size index was computed for the amplitudes of each probed-frequency, grouped according to whether it was the fundamental:

$${SSI}_{3Hz}=\frac{\left[SetSize4(3Hz)-SetSize2(3Hz)\right]}{\left[SetSize4(3Hz)+SetSize2(3Hz)\right]}$$

$${SSI}_{5Hz}=\frac{\left[SetSize4(5Hz)-SetSize2(5Hz)\right]}{\left[SetSize4(5Hz)+SetSize2(5Hz)\right]}$$

or the 2^nd^ harmonic:

$${SSI}_{6Hz}=\frac{\left[SetSize4(6Hz)-SetSize2(6Hz)\right]}{\left[SetSize4(6Hz)+SetSize2(6Hz)\right]}$$

$${SSI}_{10Hz}=\frac{\left[SetSize4(10Hz)-SetSize2(10Hz)\right]}{\left[SetSize4(10Hz)+SetSize2(10Hz)\right]}$$

These normalizing-indices allow us to combine the 3Hz and 5Hz data while compensating for intrinsic differences in frequency specific amplitudes often observed with EEG data. This was done separately for the fundamental frequencies (*SSI*_*1f*_): 3hz and 5hz index values were averaged together as were the indices for the 2^nd^ harmonics (*SSI*_*2f*_):

$${SSI}_{1f}=\frac{{SSI}_{3Hz}+{SSI}_{5Hz}}{2}$$

$${SSI}_{2f}=\frac{{SSI}_{6Hz}+{SSI}_{10Hz}}{2}$$

An index value greater than zero indicates that the neural response to the to-be-probed items are greater in set size 4 conditions than set size 2. Conversely, negative indices indicate greater neural responses in the set size 2 arrays. Index values were then averaged across subjects and subjected to statistical analyses.

At the group level, one sample t-tests (α = 0.05) were performed at each electrode to evaluate whether the indices were significantly non-zero. We applied a False Discovery Rate Correction (q = 0.10) to account for multiple comparisons.

### Induced power frequency analysis

To examine induced effects of set-size, we applied the Fourier transform to each individual trial and computed the amplitude of response at each frequency. For each frequency we then averaged the amplitudes across trials (excluding phase). Doing so allows for the extraction of induced oscillatory activity that would otherwise cancel out due to variations in phase [49]. As before, to account for uneven numbers of trials in each condition, we applied a permutation analysis using 25,000 iterations (the smaller number of iterations in the induced power analysis relative to the evoked power analysis was due to the increased computational load of performing a Fourier analysis on individual trials as opposed to the average of those trials). This process resulted in 4 averaged frequency-domain amplitude spectra computed at each electrode for each participant. Each spectra consisted of the induced power at frequencies ranging from 1–500 Hz corresponding to the encoding period. Next, we isolated amplitudes for frequencies between 1–100 Hz. These amplitudes were normalized by creating an index value for each frequency (1–100 Hz) across the 3 Hz and 5 Hz trials and subsequently averaged together (3 Hz & 5 Hz). A sample index value is shown below

$${SSI}_{4Hz}=\frac{\left[SetSize4(4Hz)-SetSize2(4Hz)\right]}{\left[SetSize4(4Hz)+SetSize2(4Hz)\right]}$$

These normalizing-indices allow us to again combine data across frequency while compensating for intrinsic differences in frequency specific amplitudes observed in EEG data. Next, the index values were averaged across the following frequency bands: delta (1 Hz—4 Hz), theta (5 Hz—7 Hz), alpha (8 Hz -12 Hz), beta (13 Hz—31 Hz) and gamma (32 Hz—100 Hz).

At the group level, one sample t-tests (α = 0.05) were performed at each electrode to evaluate whether the indices were significantly non-zero. We applied a False Discovery Rate Correction (q = 0.10) to account for multiple comparisons.

## Results

### Behavioral Accuracy

**Fig 2:**
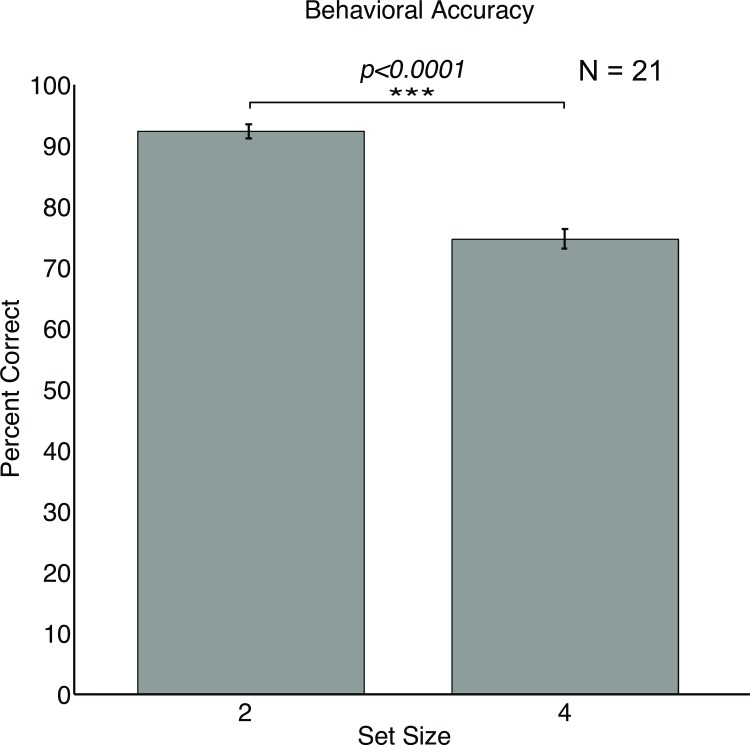
Behavioral accuracy for the set size 2 and set size 4 conditions. Participants performed significantly better on set size 2 condition. Error bars represent standard errors of the means.

First, we examined the behavioral performance in the VWM task to make sure we replicated the set size effect. As expected, accuracy was significantly better in the set size 2 condition (*M = 92*.*32*, *SD = 5*.*28)* compared to the set size 4 condition *(M = 74*.*62*, *SD = 6*.*96; t(20) = 4*.*83*, *p <0*.*0001);* see Fig 2. Secondly, we wanted to ensure that behavioral performance was not affected by differences in the flicker frequency of the probed items. In the set size 2 condition, a paired samples t-test yielded no significant difference between the 3 Hz (*M = 93*.*04*, *SD = 4*.*31)* and 5 Hz trials (*M = 91*.*59*, *SD = 4*.*49;* t(20) = 1.39, p = 0.18). In the set size 4 condition, a repeated measures ANOVA showed no significant differences between the 3 Hz (*M = 71*.*67*, *SD = 5*.*46)*, 5 Hz (*M = 75*.*68*, *SD = 6*.*17)*, 12 Hz (*M = 67*.*35*, *SD = 6*.*07)*, and 20 Hz trials (*M = 73*.*25*, *SD = 6*.*59; F(3*, *80) = 0*.*67*, *p = 0*.*57)*.

### Analysis of frequency tagging amplitude and set size effects

**Fig 3:**
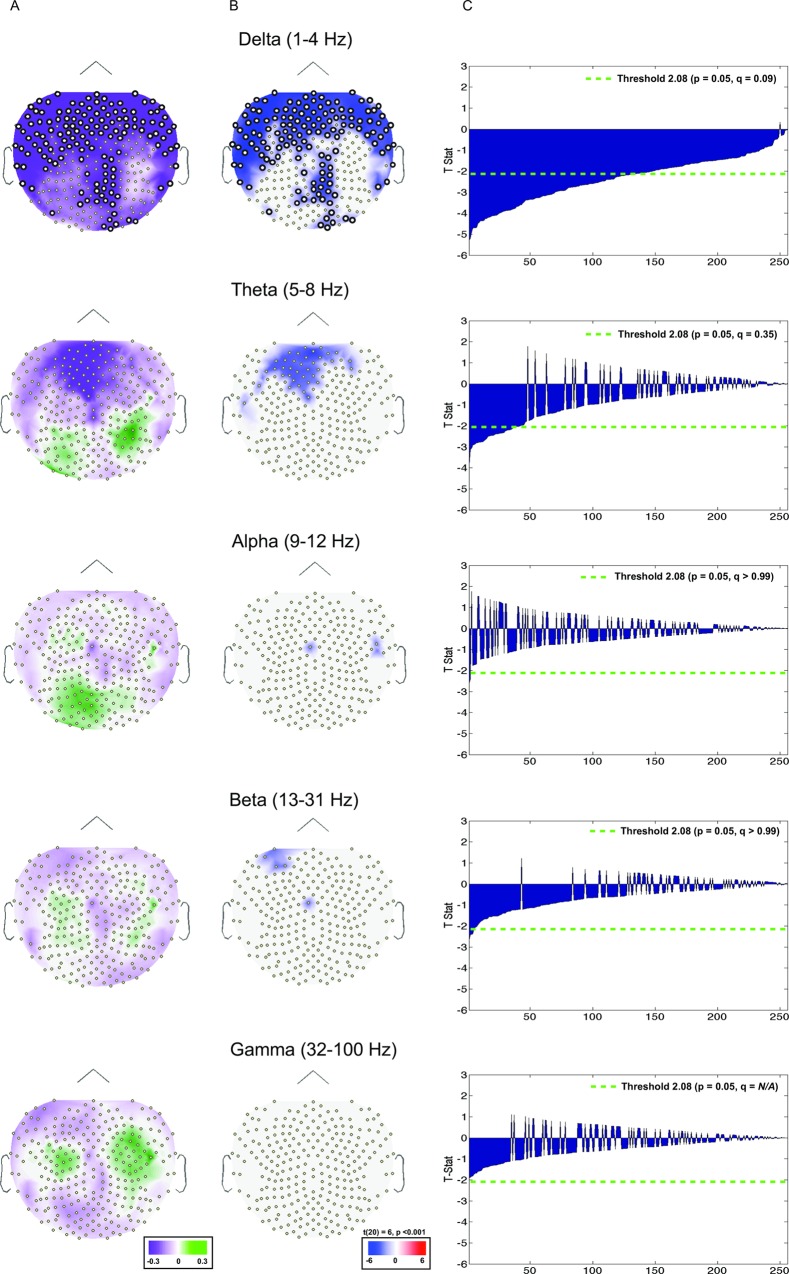
Index values and T-stats depicting set size effects (2 vs. 4) at encoding. (A) Index values (described in Materials and methods section: Frequency-tagging (evoked) analysis) plotted on topographic maps. Green corresponds to set size 4 > set size 2, purple corresponds to set size 2 > set size 4. (B) t-stats plotted on topographic maps. Plotted values correspond to t-stats at or below a *p* value of 0.05. Red corresponds to set size 4 > set size 2, blue corresponds to set size 2 > set size 4. (C) t-stats at each electrode arranged by p values. Green dotted line represents a threshold of α = 0.05. Red line represents an FDR corrected threshold for *q = 0*.*1* (see results section: Analysis of frequency tagging amplitude and set size effects). Channels significant at or above the FDR threshold are represented by a thicker border.

In Fig 3A, index values for *SSI*_1f_ and *SSI*_2f_ (see Materials and methods section: Frequency-tagging (evoked) analysis) for description) are plotted on topographic maps to illustrate set size effects at encoding for set size 2 > 4 and 4 > 2 [54]. One-sample t-tests were computed for each index across all 256 channels. The subsequent t-statistics are plotted on topographic maps in Fig 3B for both set size 2 > 4 and set size 4 > 2. For these maps, only the t-statistics that were significant at the uncorrected *p* < 0.05 level are plotted. In Fig 3C, the t-stats are ranked by *p*-value and plotted for every electrode. Positive values depict greater activity for set size 4 and negative values depict greater activity for set size 2. Green dotted lines represent a threshold equivalent to: α = 0.05. Red-dotted lines represent a FDR corrected threshold of *q* = 0.1.

We highlight the following observations. First, the indices shown in Fig 3A reveal a set of electrodes at central and posterior sites for which set size 4 yields larger responses primarily in the fundamental frequency responses. However, few of these indices are statistically significant, particularly when taking multiple comparisons into consideration (Fig 3B and 3C). Second, another set of electrodes located primarily at frontal electrode sites for which set size 2 yield larger responses in both the fundamental frequency and 2^nd^ harmonic, many of these indices are statistically significant, even when taking multiple comparisons into consideration (Fig 3B and 3C).

### Analysis of induced power and set size effects

**Fig 4:**
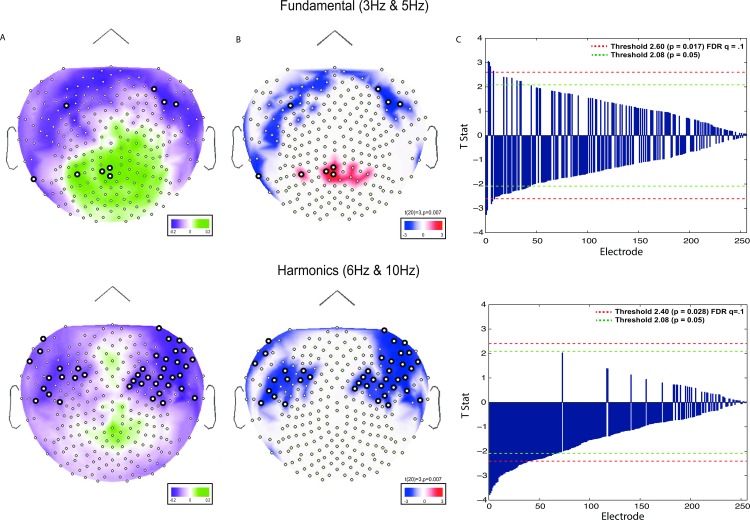
Index values and T-stats depicting induced power set size effects (2 vs. 4) at encoding. (A) Index values (described Materials and methods section: Induced power frequency analysis) plotted on topographic maps. Green corresponds to set size 4 > set size 2, purple corresponds to set size 2 > set size 4. (B) t-stats plotted on topographic maps. Plotted values correspond t-stats at or below a p value of 0.05. Red corresponds to set size 4 > set size 2, blue corresponds to set size 2 > set size 4. (C) t-stats at each electrode arranged by p values. Green dotted line represents a threshold of α = 0.05. Channels significant at or above the FDR threshold are represented by a thicker border. Each row depicts data from a single frequency band: delta (top), theta, alpha, beta, gamma (bottom).

Index values for five frequency bands (beta, theta, alpha, beta, gamma) are plotted on topographic maps to illustrate induced power set size effects at encoding (Fig 4A). One-sample t-tests were computed for each index across all 256 channels and plotted as a function of set size 2 > 4 and set size 4 > 2 (Fig 4B). Only the t-statistics that were significant at the uncorrected *p* < 0.05 level are plotted. As before, the t-stats were organized by p-value and plotted for every electrode following the conventions described above (Fig 4C: positive: set size 4 > 2; negative: set size 2 > 4). Green dotted lines represent a threshold equivalent to α = 0.05. For the delta band, the 0.05 alpha value corresponds to an FDR q-value of 0.09. In contrast, few of the t-stats for the theta, alpha and beta frequency bands are significant at α = 0.05 (FDR *qs* > 0.35, 0.99 & 0.99, respectively). No significant t-stats were observed for the gamma frequency band.

We highlight the following observations. Increased delta power in the set size 2 > set size 4 was observed in frontal and posterior electrode cites. Although fewer electrodes reached statistical significance, some increased theta activity was observed in the set size 2 condition relative to the set size 4 condition, primarily at frontal locations. However, it should be noted that these electrodes did not survive FDR correction.

## Discussion

To better understand the neural basis of VWM capacity limitations we investigated encoding-related processing differences as a function of set size and retrieval success. To examine potentially stimulus-specific neural correlates of set-size we leveraged the classic EEG frequency-tagging approach. To investigate potentially task-related neural correlates of set-size we examined changes in induced power.

Frequency-tagging involves flickering each stimulus at a unique frequency and measuring the evoked EEG power at the corresponding frequency. Index values were computed allowing a comparison of frequency-tags across the set size 2 and set size 4 conditions. These data revealed that over anterior sites above frontal cortex, the frequency-tags, especially for the 2^nd^ harmonic, were significantly larger in the set size 2 condition compared to the set size 4 condition. This same frontal area and additional sites over posterior regions showed induced power changes in the delta band during the VWM encoding period as a function of set size. These results indicate that VWM set size effects are partially mediated by encoding related processes. One reason why these results were observed primarily over the frontal cortex (as opposed to occipital cortex) may be related to the fact that spatial maps are highly lateralized in the early visual areas but become less lateralized as visual information reaches the frontal lobes. Since items in the set size 2 condition were presented at random locations (either bilaterally or unilaterally) and all correct trials were subsequently averaged together, this may explain the lack of set size effects over posterior electrodes.

These data extend a growing literature honing in on the neural correlates of VWM capacity. Considerable recent interest has focused on VWM maintenance. Previous fMRI studies uncovered a parametric increase in posterior parietal cortex (PPC) as a function of increased VWM set size [52,55]. For each individual, when the number of stored items exceeded working memory capacity, the BOLD signal plateaued [18]. These results suggest the presence of a finite neural resource that is depleted. The EEG literature has produced analogous results using the CDA, which increases with VWM set size up to an individual’s capacity limit [56,22].

The current results of the frequency-tagging (evoked) analysis may appear contradictory to fMRI and EEG literature reporting increased neural measures corresponding with increased set size because we report greater activity for the smaller set size condition. There is a key difference in viewing and interpreting these data comprehensively. Previous work examined the *aggregate* neural resources afforded all stimulus items. Here, the frequency-tagging approach permits measurement of individual stimuli. Thus, the aggregate response increases with set size, whereas the individual response data shows that the amount of neural resources allocated to any *single* stimulus item actually decreases. In other words, when more stimuli are presented, more resources are needed to represent them effectively in VWM, yet each item gets a smaller portion of these resources.

There is an essential role of attention as a selection process for further processing and entry into VWM. Indeed, attentional cueing reveals the large storage of iconic memory [6], and the decay in resolution between iconic and the proposed fragile and robust stages of VWM [57]. Attention facilitates performance on change detection tasks [58] and in VWM more generally [59,60]. Research on object tracking also shows important parallels between the deployment of attention in visual tasks and VWM capacity limitations. Pylyshyn and Storm [61] showed that participants track no more than 5 independently moving objects and that there exists an inverse relationship between accuracy and number of targets being tracked. Furthermore, there is evidence that working memory capacity is predictive of an individual’s tracking capacity [62]. A study by Drew & Vogel [63] showed that the capacity limitations associated with object tracking tasks appear to arise during the initial selection process when subjects are allocating their attentional resources towards the to-be-tracked items.

Additional analogs between object tracking and VWM have come from fMRI studies examining BOLD activation and the effects of attentional load. Specifically, as the number of items being tracked increases there is a corresponding linear increase in PPC activity [64,65] much like the effects observed during the maintenance phase of VWM tasks [17,19]. We note though that attention alone is insufficient to ensure successful VWM encoding. It is likely the case that encoding-specific processes are required, perhaps enabled by attention [66].

Although the effects observed in the evoked analysis were prominent for the set size 2 > set size 4 condition, it is worth mentioning that some electrodes showed the opposite effect, set size 4 > set size 2. Specifically, this was observed on 4 posterior-central electrodes at the fundamental frequency. Interestingly, these results seem to coincide with the CDA and fMRI VWM data that show increasing neural activity with increased set size [10,21]. Furthermore, enhanced connectivity has been observed between extrastriate regions and frontal and parietal sites [38–40]. Thus, one interpretation of these results can be seen as the shifting of neural resources to posterior regions given increased task demands. However, we reiterate that the majority of electrodes showed the opposite effect and advise caution in making inferences from topography to neural sources given the many known challenges associated with EEG source estimation [67].

One aspect of our results deserves further comment. The pattern of results is most evident at the 2^nd^ harmonic compared to the fundamental frequency. One possible explanation is that the first and second harmonic underlie differing cognitive processes. One suggestion is that the fundamental frequency and the second harmonic originate from different neural populations with differing topographical distributions [46]. Furthermore, the fundamental frequency is thought to be associated with low-level visual responses whereas the second harmonic is thought to reflect higher-level visual processes [44]. Subsequently, the second harmonics show greater attentional modulation compared to the fundamental frequency [46]. One assumption of the present study is that successful retrieval is contingent upon the distribution of sufficient attentional resources during encoding, thus greater signal would be expected in the second harmonic. Thus, we hypothesize that the observed differences between the fundamental frequency and the 2^nd^ harmonic are consistent with the underlying generators of the frequency-tag signal. Next, we will briefly summarize the advantages of using the frequency-tagging method for further investigating encoding related processes.

One of the major advantages of the frequency-tagging technique is that it allows for the examination of neural responses associated with specific stimuli by extracting the amplitude associated with each “frequency-tag”. However, studying VWM encoding presents a challenge in the form of disambiguating encoding from maintenance phases. This is especially a concern for fMRI research given fMRI’s poor temporal resolution and infrequent use of partial trials [16]. However, even traditional ERPs can present similar challenges because evoked EEG components evolve over tens to hundreds of milliseconds. Thus, frequency-tagging provides an ideal technique to isolate encoding related processes and to characterize the neurophysiological signal associated with individual items.

The induced frequency analysis also showed greater activity in the set size 2 condition in the delta band at frontal and posterior electrode cites as well as some theta activity in frontal electrodes. No significant differences were observed in alpha, beta or gamma bands. The theta band oscillations are consistent with previous observations that they may be driven by cortico-hippocampal interactions [68]. Theta band oscillations are associated with various cognitive domains, including working memory [69], episodic memory encoding and retrieval [47], cognitive control [70], selective attention [71], and attentional allocation to target stimuli [72]. However, we remind the reader that although some electrodes in the theta band were significant at an alpha of 0.05, none of the electrodes survived FDR correction. Thus, the evidence for the involvement of theta activity in the current task is minimal at best. Less is known regarding the role of delta oscillations in human cognition [73]. Some evidence suggests there are functional similarities between the delta and theta bands specifically as it relates to cognitive control [74]. Given its slow oscillation, the delta band is well suited for long-range communication and may serve as a mechanism by which frontal lobes modulate distant brain regions [73]. Delta oscillations contribute to the P300 oddball response and are modulated by novel distractors as well as task switching cues [75]. Delta band oscillations have also been associated with attentional selection and stimulus expectancy [76, 77]. Finally, as it relates to VWM, studies suggest that delta oscillations increase in regions that relate to inhibition of interference during the task [73,78,79].

On a related note, the set size 2 condition contained two squares flickering at 12 Hz and 20 Hz in addition to the to-be-remembered stimuli. These squares were added so as to avoid any potential artifacts stemming from more flickering stimuli in the set size 4 condition. Participants were instructed to ignore these flickering squares and, the significantly better behavioral performance in the set size 2 condition indicates they did so successfully. Thus, solely the set size 2 condition required the filtering of task-irrelevant distractors whose spatial location differed between trials. Thus, based on the studies reviewed in the previous sections [71–73,78,79], a speculative interpretation of the induced delta and theta power is that these frequencies may be relevant in distractor filtering and attentional selection.

In closing, by examining the neural signals generated by frequency tags for each to-be-encoded stimulus, we build upon our previous findings [45] that highlighted the role of errors at encoding as a contributing factor of VWM capacity. By focusing on neural set-size effects for correct trials, the current study revealed that like VWM as a whole, encoding-related neural resources are likely capacity-limited. These resources get distributed across to-be-encoded items such that the amount of resources allocated to each item is inversely proportional to the number of items being encoded. We conclude that this encoding-specific capacity limitation can account at least in part for declines in behavioral performance on VWM tasks observed as set sizes increase. These findings further highlight the importance of encoding-stage processes in constraining models of VWM capacity.
