# Correction: Induced and Evoked Human Electrophysiological Correlates of Visual Working Memory Set-Size Effects at Encoding

**DOI:** 10.1371/journal.pone.0173650

**Published:** 2017-03-06

**Authors:** Gennadiy Gurariy, Kyle W. Killebrew, Marian E. Berryhill, Gideon P. Caplovitz

The images for Figs 3 and 4 are incorrectly switched. The image that appears as Fig 3 should be Fig 4, and the image that appears as Fig 4 should be Fig 3. The figure captions appear in the correct order.

**Fig 3:**
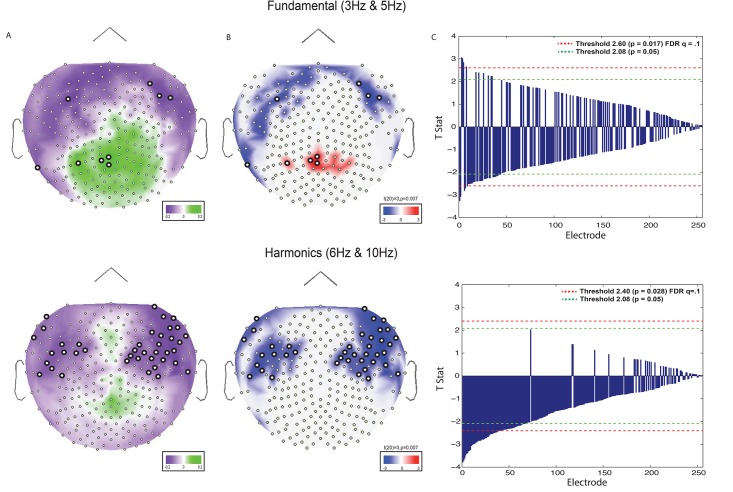
Index values and T-stats depicting set size effects (2 vs. 4) at encoding. (A) Index values (described in Materials and methods section: Frequency-tagging (evoked) analysis) plotted on topographic maps. Green corresponds to set size 4 > set size 2, purple corresponds to set size 2 > set size 4. (B) t-stats plotted on topographic maps. Plotted values correspond to t-stats at or below a *p* value of 0.05. Red corresponds to set size 4 > set size 2, blue corresponds to set size 2 > set size 4. (C) t-stats at each electrode arranged by p values. Green dotted line represents a threshold of α = 0.05. Red line represents an FDR corrected threshold for *q = 0*.*1* (see results section: Analysis of frequency tagging amplitude and set size effects). Channels significant at or above the FDR threshold are represented by a thicker border.

**Fig 4:**
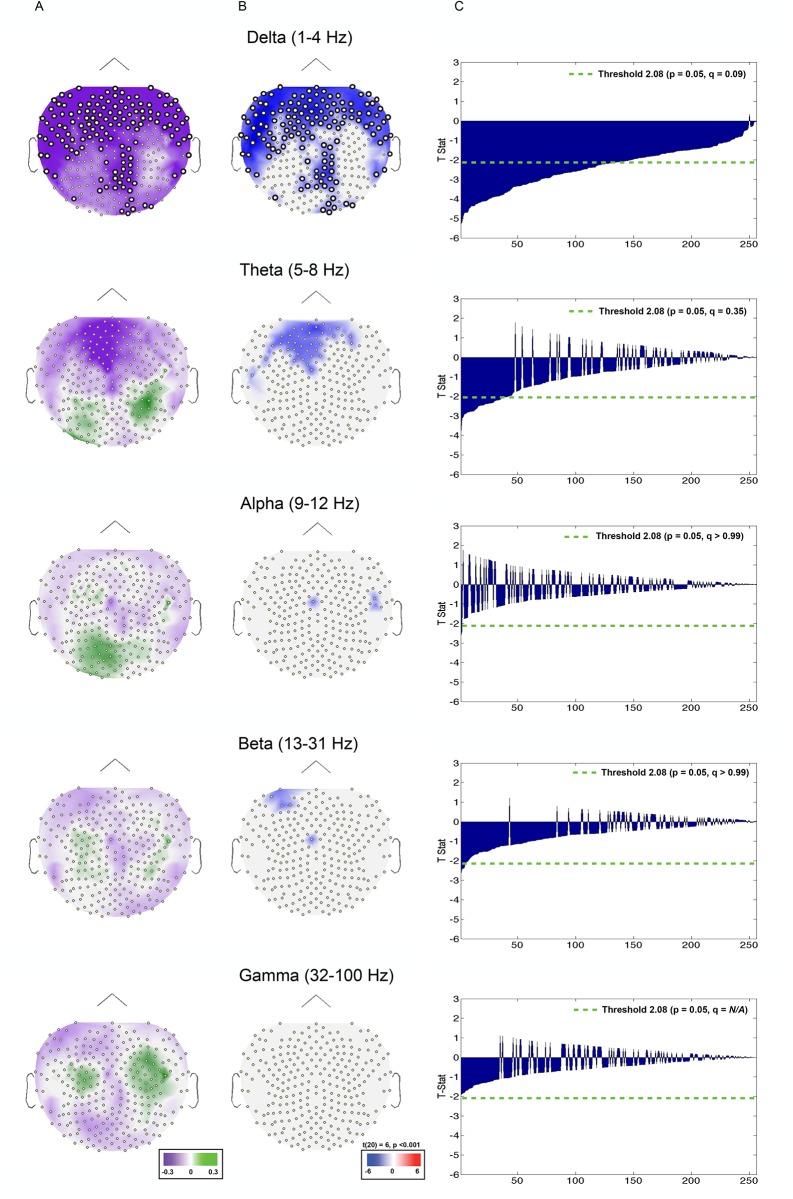
Index values and T-stats depicting induced power set size effects (2 vs. 4) at encoding. (A) Index values (described Materials and methods section: Induced power frequency analysis) plotted on topographic maps. Green corresponds to set size 4 > set size 2, purple corresponds to set size 2 > set size 4. (B) t-stats plotted on topographic maps. Plotted values correspond t-stats at or below a p value of 0.05. Red corresponds to set size 4 > set size 2, blue corresponds to set size 2 > set size 4. (C) t-stats at each electrode arranged by p values. Green dotted line represents a threshold of α = 0.05. Channels significant at or above the FDR threshold are represented by a thicker border. Each row depicts data from a single frequency band: delta (top), theta, alpha, beta, gamma (bottom).
